# Retraction: An eel-like robot based on a dielectric elastomer

**DOI:** 10.1371/journal.pone.0344428

**Published:** 2026-04-02

**Authors:** The PLOS One Editors

After this article [1] was published, the author requested retraction due to errors that may impact the results and conclusions. Specifically, Formula 7 in [1] contains an incorrect minus sign, which should be a plus sign. The author stated that this error directly affects the subsequent data, as the formula is used to calculate key parameters related to the eel-like robot’s performance. They also stated that propulsion speed data in Fig 11 were derived from these calculations, and that the conclusions about the robot’s effectiveness, optimal operating conditions, and performance under specific parameters are no longer supported.

The *PLOS One* Editors reviewed the author’s request in consultation with an independent member of the *PLOS One* Editorial Board and note that [1] indicates that propulsion speed, as reported in Fig 11, was calculated using marker position data extracted from video of the robot swimming (Fig 10 of [1], ‘Measurement method of swimming speed’). The *PLOS One* Editors were unable to obtain the underlying data for [1], including the raw images and videos of the eel robot in motion from which kinematics were extracted. In the absence of raw videos of the robot in motion, the consulting member of the *PLOS One* Editorial Board concluded that questions about the validity of the results and conclusions related to swimming speed and whether the robot movement followed Formula 7 in [1] could not be fully resolved.

The member of the *PLOS One* Editorial Board also stated that the analysis on the number of swims associated with the ‘robot swim’ number, including Fig 13, is incorrect. They stated that the ‘robot swim’ number appears to be an attempt to calculate the Strouhal number [2], and therefore the ‘robot swim’ number is not correctly defined in [1]. They also stated that Section 5.2 Number of swims, and the corresponding parts of the Discussion, are incorrect.

In light of the author’s request, the above concerns, and the absence of the data underlying [1], the *PLOS One* Editors no longer have confidence in the results and conclusions in [1], and therefore retract this article.

The author agreed with the retraction

Fig 1 in [1] was previously published in [3] under a CC BY 4.0 license. No changes were made to the reused content. Fig 1 is subject to the license that applies to the original article
